# The role of anti-malarial drugs in eliminating malaria

**DOI:** 10.1186/1475-2875-7-S1-S8

**Published:** 2008-12-11

**Authors:** Nicholas J White

**Affiliations:** 1 Mahidol – Oxford Tropical Medicine Research Unit, Faculty of Tropical Medicine, Mahidol University, 420/6 Rajvithi Road, Bangkok 10400, Thailand; 2 Centre for Clinical Vaccinology and Tropical Medicine, Churchill Hospital, Oxford, UK

## Abstract

Effective anti-malarial drug treatment reduces malaria transmission. This alone can reduce the incidence and prevalence of malaria, although the effects are greater in areas of low transmission where a greater proportion of the infectious reservoir is symptomatic and receives anti-malarial treatment. Effective treatment has greater effects on the transmission of falciparum malaria, where gametocytogenesis is delayed, compared with the other human malarias in which peak gametocytaemia and transmissibility coincides with peak asexual parasite densities. Mature *Plasmodium falciparum *gametocytes are more drug resistant and affected only by artemisinins and 8-aminoquinolines. The key operational question now is whether primaquine should be added to artemisinin combination treatments for the treatment of falciparum malaria to reduce further the transmissibility of the treated infection. Radical treatment with primaquine plays a key role in the eradication of vivax and ovale malaria. More evidence is needed on the safety of primaquine when administered without screening for G6PD deficiency to inform individual and mass treatment approaches in the context of malaria elimination programmes.

## Background

Anti-malarial drugs play a central role in the control and ultimate elimination of malaria, but, in most circumstances, they cannot do the job alone. Whether malaria can be eliminated with current tools is a subject of active debate [1], but it is generally agreed that the tools to reduce substantially the global burden of malaria are available. The corollary is that if the current classes of effective anti-malarial drugs (notably the artemisinin derivatives) are lost, then effective control and elimination will not be possible. Anti-malarials, if effective, reduce the transmission of malaria, but the relationship between efficacy and transmission reduction is not straightforward. This relationship is the subject of this review.

### Epidemiological considerations

Where transmission of malaria is intense, even highly effective interventions which reduce mortality may have little noticeable effect on the clinical pattern of malaria. A 95% reduction in transmission from 500 infective bites per year to 25 will not change the incidence or prevalence of malaria noticeably. In this context, there is a tremendous redundancy in the transmission reservoir (from asymptomatic gametocyte carriers), and the use of effective anti-malarial drugs will have relatively little impact on transmission intensity. For example, in a high transmission setting in Western Kenya, it was estimated, based on mosquito feeding studies, that 28% of all transmission derived from children ≥10 years and adults – a largely asymptomatic population [2]. This would correspond to 10–50 infectious bites per year, plenty to sustain a hyper or holoendemic pattern of malaria even if every symptomatic patient received highly effective drugs. But as transmission intensity falls, the premunition of the population declines, and an increasing proportion of transmission derives from symptomatic individuals, who will seek anti-malarial treatment. In a detailed epidemiological study conducted in a low transmission setting on the Thai-Burmese border (entomological inoculation rate <1 per species) all *P. falciparum *infections were symptomatic, but approximately 10% of *Plasmodium vivax *infections were still asymptomatic [3]. Although this is probably representative of most low transmission settings, it is important to note that transmission intensity is remarkably heterogeneous over short geographic distances, and that low transmission areas usually contain small foci of much higher transmission intensity which act as reservoirs of infection. Inhabitants of these small foci sustain malaria over the annual dry season. Premunition does not change abruptly, so if there are sudden decreases (or increases) in transmission intensity then the changes in clinical epidemiology lag behind (hysteresis). Thus, the effects of anti-malarial treatment on the incidence and prevalence of malaria increase as transmission falls. Unfortunately, there are no precise estimates of this relationship.

### Biological considerations

As human malaria is transmitted by sexual stages of the parasites, not asexual stages, infecting anopheline mosquito vectors, transmission depends upon the duration for which gametocytes are carried in the blood, the infectivity of this gametocytaemia to the local vectors, and the abundance and behaviour of the vectors [4]. The attractiveness of humans to mosquitoes is unevenly distributed amongst the human population. Mosquitoes apparently prefer to bite individuals with lower biomass and lower body surface area [5], pregnant women [6], and those with smelly feet [7,8], amongst other things. Gametocytaemia is detectable by microscopy down to densities of approximately 10–20/uL. At least one male gametocyte's progeny (eight microgametes) and one female macrogamete are required in a mosquito blood meal (approx 2–3 μL) for infection to occur. Thus, gametocyte densities of 1/μL can theoretically infect vector mosquitoes [4,9]. This is below the density which can be detected by routine microscopy. It has sometimes been stated that malaria can be transmitted (to mosquitoes) by individuals who are not carrying gametocytes. This is impossible. What is actually meant is that the individuals obviously were carrying viable gametocytes in their blood, but the density was below that detectable by microscopy [10]. As the progeny of at least two gametes are required to form a zygote, the probabilities of transmission as gametocyte densities fall below 1/μL rapidly become vanishingly small. Whether gametocytes concentrate in the dermal capillaries (as they ought to!) still remains unclear, but even if they do, these numerical considerations still apply. *Plasmodium falciparum *differs from the other three human malarias in two important respects; first, gametocyte formation is delayed with respect to the peak production of asexual stages [11-13], and, second, that mature gametocytes are resistant to most of the anti-malarial drugs, which affect asexual stages. The developing sexual stages of *P. falciparum *(stages I to IV) remain sequestered in the microvasculature for approximately 10 days before appearing as morphologically distinct male and female gametocytes (stage 5) in the peripheral blood. Peak transmissibility coincides approximately with peak gametocytaemia [12]. Several factors increase the production of gametocytes in falciparum malaria, most of which can be considered under the general rubric of "stress"; these include long duration of infection, anaemia, partially effective immune responses, and partially effective drugs [14-17].

In infections with *P. vivax, P. malariae *and *P. ovale*, the asexual and sexual stages appear almost together [18-20], and, in contrast to *P. falciparum *[19], are sensitive to all the drugs which kill the asexual stages [21] These three malaria parasites transmit very efficiently at low parasite densities – much more efficiently than *P. falciparum*. Thus, a greater proportion of infected subjects without detectable gametocytaemia can transmit these malaria infections. Because the majority of patients presenting with these so-called benign malarias are already infective to biting anopheline mosquito vectors when they present ill for treatment, administration of effective anti-malarial drugs has less effect on overall transmission compared with falciparum malaria.

### Pharmacological considerations

All anti-malarial drugs which kill asexual stages also kill the early stages of *P. falciparum *gametocytes, as well as the mature *P. vivax, malariae*, and *ovale *gametocytes. Thus the combination of reducing the asexual stage progenitors of the sexual stages, and killing the gametocytes themselves reduces transmissibility. In general, the asexual and sexual stage activities parallel each other [21-24], although the quantitative relationships between anti-malarial drug concentrations (pharmacokinetics; PK) and reduction in transmissibility (pharmacodynamics; PD) are not well characterized [25], particularly for vivax, malariae, and ovale infections. For fully sensitive asexual stage parasites, the artemisinin derivatives produce the most rapid reductions in parasitaemia [26,27], followed by chloroquine [28], then closely behind the other quinolines and related drugs, together with atovaquone, and finally the antifols. It is not clear whether amodiaquine, piperaquine and pyronaridine are closer to chloroquine than quinine and mefloquine in this batting order but if they are, the differences are small. The antibiotics with anti-malarial activity are generally less active and clear parasitaemia more slowly than the anti-malarial drugs [23]. At a population level there is wide inter-individual variation in the effects of a single anti-malarial treatment on the reduction in parasitaemia. This derives from host differences in anti-malarial adherence, absorption, distribution (drug elimination is less relevant), and splenic function as well as differences in the numbers, stage, synchronicity and susceptibility of the infecting parasites [26]. Anti-malarial drug susceptibility of the infecting parasites becomes important when the drug concentrations in blood do not produce the maximum effect (i.e. are below the minimum parasiticidal concentration; MPC). The proportion of treated patients with blood concentrations below the MPC is a function of drug quality, dose taken, host pharmacokinetics, and resistance. As resistance worsens this proportion increases [29].

The overall effects of anti-malarial drugs on *P. falciparum *gametocytaemia (i.e. late stage gametocytes which circulate and are detectable by microscopy) is a composite of their effects on the progenitors, the early (more drug susceptible) stage 1 to 3 sequestered gametocytes, and the more mature (less drug susceptible) stages. *P. falciparum *gametocytaemia following antifol treatment (best documented for sulphadoxine-pyrimethamine) is consistently greater than that following other drug classes [4,30,31], whereas treatment with the artemisinin derivatives is associated with lower rates of gametocyte carriage [13,14,27,32,33]. The 8-aminoquinolines (primaquine, quinocide, tafenoquine) occupy a unique position. Most data refer to primaquine, the only generally available compound. Primaquine has reasonable asexual stage activity against *P. vivax *malaria [34], kill hypnozoites of *P. vivax *and *P.ovale *[35,36], and mature gametocytes of *P. falciparum *[37,38], but importantly lacks useful activity against asexual stages (and presumably early gametocytes) of *P. falciparum *[37,38]. It is not known how primaquine works in any of these three actions, nor which of its many metabolites is the active moiety. The considerable variations in immunity, pharmacokinetics and pharmacodynamics results in marked variability in transmissibility related to the activity of anti-malarial drugs [39-43].

### Anti-malarial drug resistance and transmissibility

In considering anti-malarial drug effects on transmissibility, three different components need to be considered; a) activity against asexual stages and early gametocytes, b) activity against mature infectious gametocytes, and c) sporontocidal effects in the mosquito. As drug activity against the asexual stage activity falls because of worsening anti-malarial resistance, the rate at which parasitaemia is reduced falls, and treatment failure rates increase. Gametocytaemia is a very sensitive measure of worsening drug resistance. As SP resistance worsened in South Africa, increasing gametocytaemia was the first warning sign. Importantly it preceded measurable changes in parasite clearance or a decline in cure rates [44]. Thus, transmission, and in particular transmission of resistance, would have increased before detectable changes in treatment failure rates. Okell *et al *have recently pooled data from 3,174 patients enrolled in six anti-malarial trials conducted in The Gambia and Kenya. ACT treatment (either artesunate-SP, artesunate-chloroquine or artemether lumefantrine) was associated with a significant reduction in the probability of being gametocytaemic on the day of transmission experiments (OR 0.20 95% CI 0.16–0.26), transmission to mosquitoes by slide-positive gametocyte carriers (OR mosquito infection 0.49 95% CI 0.33–0.73), and AUC of gametocyte density (ratio of means 0.35 95% CI 0.31–0.41) [45]. Partially effective anti-malarial drug treatment increases gametocyte carriage both by reducing asexual parasite killing and by providing a stress on the surviving asexual parasite population [16,17]. These factors allow a greater fraction of asexual parasites to switch to gametocyte development. The chance of an infection recrudescing also increases. Recrudescent infections are more likely than primary infections to be gametocytaemic at presentation (because of the extended duration of infection, drug "stress" and anaemia), and are also more likely to fail subsequent treatment. The net result of the increased gametocyte carriage of the primary infections, and any subsequent recrudescences, is much greater gametocyte carriage in infections caused by resistant parasites. This translates (with considerable inter-individual variation) into increased transmissibility. It is this transmission advantage that drives the spread of resistance.

For antifols and atovaquone, there are also effects in the mosquito to consider. These drugs prevent the formation of sporozoites by interfering with oocyst development in the anopheline mosquito[46,47]. Tafenoquine also has sporontocidal activity and is more active than primaquine which has little or no activity [48]. Antifol resistant parasites are more transmissible than antifol sensitive parasites in the presence of drug indicating a further location of selective pressure driving the spread of antifol resistance [49].

It is evident that anti-malarial drug resistance spreads because of the greater transmission potential of the resistant parasites in the presence of the anti-malarial drug. Most currently available anti-malarial drugs are eliminated slowly from the body and so, if they are used intensely in a malaria endemic area, a significant proportion of the community has variable concentrations of the drug in their blood. These concentrations act as a selective filter favouring the establishment of resistant infections. The degree of selection depends on several factors including the intensity of transmission, the immunity profile of the host and the pharmacokinetic and pharmacodynamic properties of the anti-malarial drug.

### Benefits of reducing transmission

Intense malaria is associated with a considerable burden of morbidity and mortality in childhood. Infant mortality rates are positively correlated with transmission intensity measured by the EIR (estimated entomological inoculation rate), although the exact relationship is not well defined [50]. Mosquitoes may bite infants proportionally more than adults; they have been shown to bite preferentially individuals with lower biomass and lower body surface [5,50]. Deployment of effective anti-malarial drugs either alone or in combination with other control measures, particularly insecticide treated bed nets and indoor residual spraying, reduces malaria morbidity and mortality [51-53]. Indeed, the introduction of effective malaria control reduces the mortality rate more than would be expected by the direct prevention of the malaria deaths alone. This is explained by many of the conditions leading to infant deaths being only indirectly or cumulatively related to malaria, such as malaria anaemia and low birth weight due to placental malaria. Importantly reducing infant mortality does not seem to be balanced by higher mortality later on [54]. Thus, there is overwhelming evidence for a benefit from reducing malaria transmission.

### Operational considerations

Artemisinin combination treatments are the recommended first-line drugs for the treatment of falciparum malaria in endemic areas. They are highly effective and well tolerated, but despite considerable increases in recent support for anti-malarial drugs, the majority of those that need ACTs do not receive them. Scaling up deployment of ACTs and insecticide-treated nets, and importantly subsidizing ACTs so that the treatment is affordable, and donating insecticide-treated nets, will have a major impact on malaria morbidity and mortality. Indoor residual spraying also has a major role to play in some areas. Should elimination programmes do anything different? The outstanding unresolved question for treatment, is whether a single "gametocytocidal" dose of primaquine (0.5 to 0.75 mg base/kg) should be added to ACT for falciparum malaria treatment [55]. Addition of a single dose of primaquine to first-line treatment has been recommended by malaria control programmes in some areas, but not others, for many years. Despite this there is remarkably little evidence on either safety or efficacy. Prevention of relapse in vivax and ovale infections, and thus control of transmission, depends on taking a two-week radical curative regimen of primaquine (i.e. 14 days of 0.25 to 0.5 mg base/kg/day). Again this is recommended in some areas, and not in others.

Although uncertain efficacy, and the difficulty in ensuring adherence to 14-day regimens, have lowered enthusiasm for pursuing radical cure in endemic areas, the main reservations which have limited the use of primaquine concern safety. Primaquine predictably causes abdominal discomfort. The effect is dose-dependent, but considerably ameliorated by food. The 8-aminoquinolines are oxidant drugs and cause oxidant haemolysis. Methaemoglobinaemia is usual [56], but in inherited red cell enzyme deficiencies which impair defences against oxidant stress, haemolysis can be severe and occasionally life-threatening [57]. The introduction of primaquine in 1951 led to the discovery of glucose-6-phosphate dehydrogenase (G6PD) deficiency, the most common human enzyme deficiency, which affects approximately 400 million people worldwide [58]. There are some 140 different genotypes of G6PD deficiency [59,60] with corresponding phenoytypes that vary from mild to severe. The extent of oxidant haemolysis depends on the degree and nature of deficiency. Enzyme deficiency protects against severe malaria and so the abnormality is prevalent mainly in areas where malaria is or was prevalent. It is often recommended that G6PD deficiency testing be performed before administering treatment. In reality testing is usually unavailable. Several rapid tests are available in theory (but deployment is very limited) and there is insufficient evidence on their sensitivity and specificity to recommend their general deployment. The semi-quantitative fluorescence spot test is more demanding but reliable, and quantitation requires a spectrophotometric assay. So, in practice testing is usually unavailable and primaquine is often not given.

### Mass treatment

Mass treatment of the entire population with anti-malarial drugs was performed on many occasions in the eradication programmes of the 1950s and 1960s, and has been employed sporadically since then. This experience has been reviewed recently [61]. As a strategy it has not found favour in recent years. The alternative proactive approaches are mass screening and treatment (MST). This has been preferred over mass treatment in Western Cambodia in current efforts to contain and eliminate malaria there. MST is a more logistically demanding approach, and it assumes that most of the infectious reservoir will have detectable parasitaemias at the time of screening. Obviously, for *P. vivax *and *P. ovale*, screening will not detect hypnozoite carriage. The advantages and disadvantages of these two different approaches bear further examination and evaluation. Mass treatment and MST would be more acceptable if toxicity concerns over administration of the 8 aminoquinolines, without screening for G6PD deficiency, in the population can be overcome.

## Competing interests

The author is co-chairman of the WHO GMP technical expert group on the prevention and treatment of malaria.    
